# Hounsfield unit for assessing bone mineral density distribution within lumbar vertebrae and its clinical values

**DOI:** 10.3389/fendo.2024.1398367

**Published:** 2024-06-13

**Authors:** Jiabao Chen, Yanhong Li, Han Zheng, Haotian Li, Haidong Wang, Lei Ma

**Affiliations:** ^1^ Department of Spinal Surgery, The Third Hospital of Hebei Medical University, Shijiazhuang, China; ^2^ Department of Internal Medical, Hebei Medical University, Shijiazhuang, China

**Keywords:** Hounsfield unit, bone mineral density distribution, lumbar vertebrae, osteoporosis, osteoporotic vertebral compression fractures, lumbar surgery

## Abstract

**Study Design:**

Retrospective radiological analysis.

**Objective:**

The aim of this study is to evaluate the distribution of bone mineral density (BMD) in lumbar vertebrae using the Hounsfield unit (HU) measurement method and investigate the clinical implications of HU values for assessing lumbar vertebrae BMD.

**Method:**

Two hundred and ninety-six patients were retrospectively reviewed and divided into six groups according to age: Group 1(20–29 years old), Group 2 (30–39 years old), Group 3 (40–49 years old), Group 4 (50–59 years old), Group 5 (60–69 years old), Group 6 (70–79 years old). Six different locations from each vertebra of L1-L5 were selected as regions of interest: the anterior, middle and posterior parts of the upper and lower slices of the vertebrae. HU values were measured for the six regions of interest, followed by statistical analysis.

**Results:**

The HU values of vertebrae showed a decreasing trend from young patients to elderly patients in Group 1 to Group 5. There was no significant difference in HU values among different vertebrae in the same age group. In all age groups, the HU values of the anterior and posterior part of the vertebral body were significantly different from L1 to L3, with the anterior part of the vertebral body having lower HU values than the posterior part. The HU values of the anterior and posterior part of the vertebral body of L4 and L5 were statistically significant only in Group 5 and Group 6, and the HU values of the anterior part of the vertebral body were lower than those of the posterior part. The HU values of posterior part of L4 and L5 in Group6 were higher than those in Group5.

**Conclusion:**

Bone mineral density in the lumbar vertebrae is not uniformly distributed, potentially attributed to varying stress stimuli. The assessment of local HU values in the lumbar spine is of significant importance for surgical treatment.

## Introduction

Osteoporosis is a systemic bone disease due to a variety of causes (1). It is indicated by a decrease in bone density and mass, destruction of bone microstructure, and increased bone fragility. In recent years, the measurement of Hounsfield units (HU) by computed tomography (CT) has been recognized as a useful technique for assessing bone quality (2–6). The correlation between the CT HU value and bone mineral density (BMD) and compressive strength has been demonstrated, and the HU value can represent the BMD of the vertebra (7). HU values have been widely used in osteoporosis assessment with the advantage of providing BMD data within the vertebrae. Clinically, HU values can be easily obtained from CT scans without increasing patient costs.

Several studies have suggested the value of HU values in the assessment of spinal mineral density. Zou Da et al. proposed that the CT HU value of L1–4 corresponding to L1 ≤ 110, L2 ≤ 100, L3 ≤ 85 or L4 ≤ 80, respectively, could be diagnosed as osteoporosis (8). At present, the average HU value of lumbar spine has been widely used in clinical practice, such as predicting osteoporotic vertebral compression fractures and predicting cage subsidence (9–11). On this basis, we found that the BMD of the spine was not uniformly distributed. For example, in patients with osteoporosis, osteoporotic vertebral compression fractures (OVCFs) are more likely to occur in the anterior and middle part of the vertebral body. In addition, we found that a subset of patients with Dual-energy X-ray absorptiometry (DXA) indicating low bone density still had hard bone in the posterior vertebral structure.​The structure of the vertebrae is complex and the bone density of the vertebrae is not uniformly distributed. Current methods can only describe the overall BMD of the lumbar spine and still have some limitations in clinical practice. Therefore, we believe that the assessment of the lumbar BMD should be refined.

This study improved the measurement method of CT HU value of lumbar spine. HU values for multiple regions of interest were collected to explore the BMD distribution of lumbar spinal. The objectives of this study were: firstly, to explore the characteristics of cancellous BMD distribution in lumbar vertebrae based on CT HU measurements; and secondly, to investigate the clinical application of CT HU value of lumbar spine.

## Methods

### Subjects

This study was approved by the Institutional Review Board of our hospital.

Inclusion criteria: 1. Patients admitted to our hospital with degenerative lumbar disease, such as lumbar disc degeneration, lumbar disc herniation, and lumbar spinal stenosis. 2. Full lumbar anteroposterior and lateral X-rays, lumbar CT and lumbar MRI were available for measurement. 3. Age between 20 and 79 years old.

Exclusion criteria: 1. Spinal instability, lumbar spondylolisthesis, previous lumbar surgery. 2. Spinal deformity and scoliosis, sagittal or coronal spinal disequilibrium. 3. Tuberculosis, tumor, fracture, inflammation, infection and other bone abnormalities. 4. Ankylosing spondylitis. 5. Long-term use of glucocorticoids.

By retrieving the medical records from January 2021 to June 2023 in our hospital, 296 patients who met both the inclusion and exclusion criteria were retrospectively reviewed, they were divided into six groups according to age: Group 1 (20–29 years old), Group 2 (30–39 years old), Group 3 (40–49 years old), Group 4 (50–59 years old), Group 5 (60–69 years old), Group 6 (70–79 years old).

### Date collection and assessment

Demographic data of the patients, including gender, age, BMI, were recorded. The imaging data were measured by two spine surgeons with more than three years of experience in imaging measurements.

The HU measurement for lumbar vertebra was obtained by using a protocol described similar to Schreiber on CT examination (2). All subjects were scanned with a 64 slice multi-detector CT scanner (Siemens Sensation 64, Erlangen, Germany) according to the following parameters: slice thickness 1.5 mm, distance 1.5 mm, tube voltage 120 kV. HU measurements were obtained from PACS (Picture Archiving and Communication Systems) Imaging System for lumbar vertebra.

**Figure 1:**
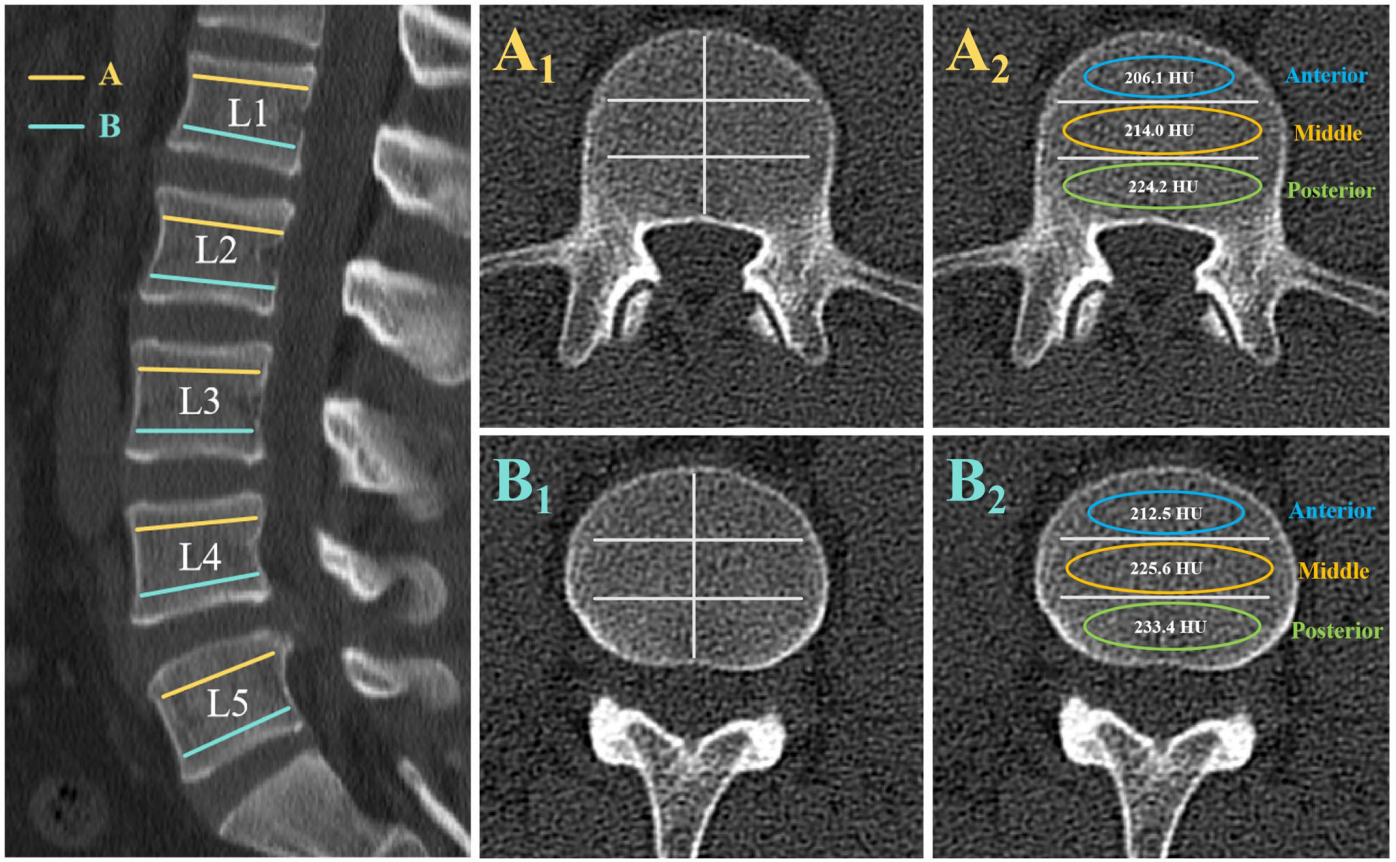
Computed tomography scan illustrating the method of determining the HU value with use of an elliptical region of interest (ROI). The left image shows the axial slices of interest on a sagittal slice of a computed tomography scan of the lumbar vertebra. Slice A was taken inferior to the superior end plate, and slice B was taken superior to the inferior end plate. At each axial slice, three different locations were selected as ROI for HU measurements: the anterior part of the vertebral body, the middle part of the vertebral body, and the posterior part of the vertebral body. Elliptical ROI were drawn as large as possible, excluding cortical edges to prevent volume averaging. The right images show the HU values generated by the imaging software program.

Two different axial slices were selected from each vertebra of L1-L5: slice A was selected inferior to the upper endplate (upper 1/2 part of the vertebra), and slice B was selected superior to the lower endplate (lower 1/2 part of the vertebra). At each axial slice, three different locations were selected as regions of interest (ROI) for HU measurements: the anterior part of the vertebral body, the middle part of the vertebral body, and the posterior part of the vertebral body. ROI was designed to include as much trabecular bone as possible, avoiding cortical bone and heterogeneous areas such as the posterior venous plexus and bone islands. (**Figure 1**) All imaging parameters were measured by two independent observers, and the average of the two measurements was collected. The average of the HU values of the six ROI within the vertebrae was used as the HU value of the vertebrae. The mean values of the anterior, middle, and posterior parts of the A and B slices were used as the HU values of the anterior, middle, and posterior parts of the vertebral body, respectively, and the statistical analysis followed.

### Statistical analysis

Data were analyzed using Statistical Product and Service Solutions software (version 26; SPSS, Chicago, IL). Continuous variables were recorded as mean ± standard deviation, and categorical variables were expressed as frequency or percentages. The interclass correlation coefficients (ICCs) were calculated to assess interobserver reliability. Analysis of Variance (ANOVA) was used to compare the HU value among multi-subgroups, and the LSD method was used for pairwise comparisons between groups. The statistical significance was set at p < 0.05.

## Results

### Patient characteristics

A total of 296 patients, 148 males and 148 females, were enrolled in the study. Included patients were grouped according to age: Group 1(20–29 years old, n=50), Group 2 (30–39 years old, n=47), Group 3 (40–49 years old, n=50), Group 4 (50–59 years old, n=50), Group 5 (60–69 years old, n=50), Group 6 (70–79 years old, n=49). There was no significant difference in BMI among the groups (**Table 1**).

**Table 1:** Comparison of general data among the six different age subgroups.

	Group 1	Group 2	Group 3	Group 4	Group 5	Group 6	*p*
Age	25.10 ± 3.03	35.45 ± 2.73	45.56 ± 2.98	54.86 ± 3.06	64.68 ± 3.20	73.94 ± 3.53	<0.001
BMI	26.75 ± 4.02	26.00 ± 4.31	25.01 ± 3.28	26.37 ± 3.63	25.80 ± 3.72	25.23 ± 3.99	0.195
No.	50	47	50	50	50	49	–
Man	25	24	25	25	25	24	–
Woman	25	23	25	25	25	25	–

### Consistency test

The inter-rater reliability of measurements obtained by two spinal surgeons was assessed using the Interclass Correlation Coefficient (ICC), which exceeded 0.98 at each location (anterior, middle, and posterior parts of the vertebral body) within the L1 to L5 vertebrae across all age groups, indicating high agreement between the data measured.

### HU value within the lumbar vertebra among age subgroups

Among the age groups, the HU values of the L1 to L5 vertebrae showed a significant difference from Group 1 to Group 5, with a decreasing trend. There was no significant difference in HU values of L1-L5 between Group 5 and Group 6. Within each age group, L1 and L5 had higher HU values while L2, L3, and L4 had relatively lower HU values, though these differences were not statistically significant (**Table 2**).

**Table 2:** HU values from L1 to L5 for six different age subgroups.

	Group 1 (50)	Group 2 (47)	Group 3 (50)	Group 4 (50)	Group 5 (50)	Group 6 (49)	F	*p*
L1	202.65 ± 32.48	188.97 ± 30.94^a^	168.31 ± 42.32^ab^	117.01 ± 33.47^abc^	93.99 ± 26.63^abcd^	96.56 ± 36.55^abcd^	97.728	<0.001
L2	197.23 ± 35.69	187.60 ± 34.27	164.14 ± 43.63^ab^	112.32 ± 38.82^abc^	90.58 ± 25.45^abcd^	86.31 ± 32.36^abcd^	95.349	<0.001
L3	199.69 + 36.98	184.86 ± 33.22^a^	159.87 ± 44.99^ab^	107.74 ± 36.35^abc^	88.19 ± 27.55^abcd^	85.84 ± 36.05^abcd^	94.383	<0.001
L4	200.60 ± 36.80	181.39 ± 36.49^a^	156.79 ± 44.12^ab^	109.35 ± 34.44^abc^	89.17 ± 28.90^abcd^	89.21 ± 40.79^abcd^	93.544	<0.001
L5	207.01 ± 40.13	186.83 ± 40.68^a^	161.22 ± 43.18^ab^	118.84 ± 36.31^abc^	98.57 ± 31.87^abcd^	99.06 ± 37.80^abcd^	72.021	<0.001
F	0.506	0.327	0.503	0.887	1.127	1.329		
p	0.731	0.860	0.733	0.472	0.344	0.260		

a p< 0.05 vs. the Group 1.

b p< 0.05 vs. the Group 2.

c p< 0.05 vs. the Group 3.

d p< 0.05 vs. the Group 4.

**Figure 2:**
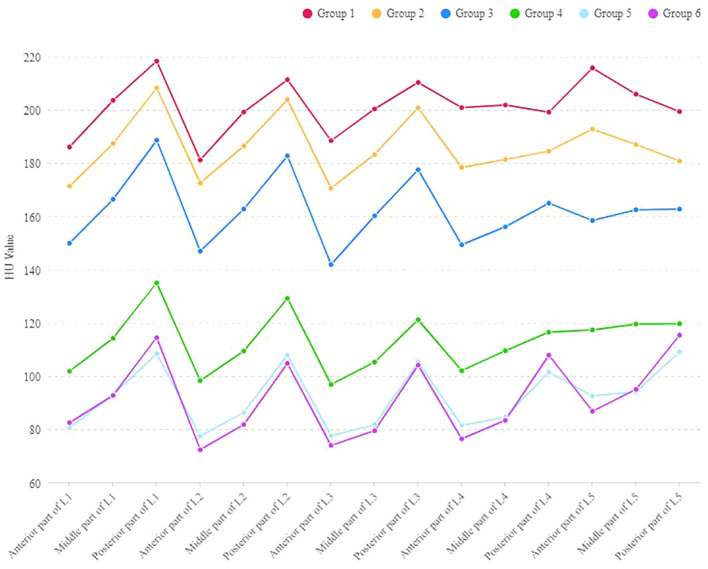
The outline of HU values at the anterior, middle and posterior parts of L1-L5 vertebral bodies in different age groups.

In each age group, the HU values of the anterior and posterior parts of L1, L2, and L3 were significantly different, with the posterior part showing higher HU values. In Group 5 and Group 6, the HU values of the posterior part of L4 and L5 were higher than the anterior part, with a statistically significant difference. However, in Group 1 to Group 4, there was no significant difference in HU values between the anterior, middle and posterior parts of the L4 and L5. In Group 1 and Group 2, the HU values in the anterior part of the L5 were higher than the posterior part, but not statistically significant (**Table 3** and **Figure 2**).

**Table 3:** HU value distribution within lumbar vertebrae from L1 to L5.

		L1	L2	L3	L4	L5
Group 1 (50)	Anterior	186.06 ± 33.08	181.17 ± 31.07	188.42 ± 38.35	200.83 ± 39.12	215.75 ± 46.67
	Middle	203.58 ± 31.67^a^	199.19 ± 34.06^a^	200.36 ± 36.79	201.82 ± 37.34	205.90 ± 40.69
	Posterior	218.31 ± 36.96^ab^	211.33 ± 45.32^a^	210.29 ± 43.05^a^	199.16 ± 44.57	199.37 ± 42.58
	*p*	<0.001	<0.001	0.024	0.946	0.168
Group 2 (47)	Anterior	171.36 ± 32.12	172.49 ± 31.93	170.56 ± 31.37	178.36 ± 38.25	192.76 ± 42.61
	Middle	187.33 ± 30.80^a^	186.46 ± 33.62	183.24 ± 34.57	181.32 ± 35.41	186.95 ± 44.06
	Posterior	208.23 ± 34.20^ab^	203.84 ± 42.75^ab^	200.79 ± 40.11^ab^	184.49 ± 42.05	180.78 ± 46.48
	*p*	<0.001	<0.001	<0.001	0.745	0.428
Group 3 (50)	Anterior	149.91 ± 41.59	146.92 ± 42.47	141.90 ± 43.04	149.33 ± 42.73	158.45 ± 45.82
	Middle	166.42 ± 43.32	162.75 ± 40.64	160.18 ± 44.31	156.09 ± 43.49	162.47 ± 42.11
	Posterior	188.61 ± 45.77^ab^	182.73 ± 50.81^ab^	177.52 ± 51.40^a^	164.94 ± 41.88	162.73 ± 49.86
	*p*	<0.001	0.001	0.001	0.242	0.873
Group 4 (50)	Anterior	101.82 ± 33.90	98.27 ± 39.16	96.85 ± 36.17	102.04 ± 33.85	117.32 ± 34.17
	Middle	114.19 ± 33.24	109.45 ± 37.66	105.24 ± 36.38	109.52 ± 34.41	119.53 ± 37.10
	Posterior	135.02 ± 38.99^ab^	129.23 ± 44.44^ab^	121.14 ± 41.15^ab^	116.49 ± 41.84	119.67 ± 44.76
	*p*	<0.001	0.001	0.006	0.150	0.944
Group 5 (50)	Anterior	80.57 ± 26.16	77.50 ± 28.36	77.56 ± 27.71	81.43 ± 32.27	92.51 ± 34.86
	Middle	93.00 ± 29.53^a^	86.32 ± 25.68	81.78 ± 25.89	84.59 ± 27.91	94.12 ± 31.34
	Posterior	108.39 ± 29.71^ab^	107.91 ± 29.32^ab^	105.23 ± 35.34^ab^	101.51 ± 33.26^ab^	109.07 ± 35.57^ab^
	*p*	<0.001	<0.001	<0.001	0.003	0.029
Group 6 (49)	Anterior	82.53 ± 37.73	72.32 ± 29.74	73.95 ± 34.22	76.40 ± 37.21	86.77 ± 35.75
	Middle	92.72 ± 36.70	81.80 ± 31.70	79.47 ± 35.56	83.35 ± 40.77	95.03 ± 38.86
	Posterior	114.44 ± 40.24^ab^	104.78 ± 39.76^ab^	104.09 ± 43.61^ab^	107.87 ± 47.83^ab^	115.38 ± 44.52^ab^
	*p*	<0.001	<0.001	<0.001	0.001	0.002

Values were expressed as the mean ± SD. Statistical analyses were conducted by one-way ANOVA, followed by LSD post hoc test.

a p < 0.05 vs. the Anterior part of vertebral body.

b p < 0.05 vs. the Middle part of vertebral body.

## Discussion

Osteoporosis is common in older individuals and postmenopausal women, increasing their risk of fractures and societal burden. Dual-energy X-ray absorptiometry (DXA) is a widely used method for assessing bone mineral density (BMD), with the World Health Organization (WHO) defining a T-score of less than -2.5 as indicative of osteoporosis. However, there are limitations to using DXA in assessing spinal BMD. As early as 2004, Shigeyuki Muraki et al. proposed that degenerative lumbar spine disease could lead to overestimation of lumbar bone density, potentially masking osteoporosis in patients (12). DXA cannot differentiate between cortical and cancellous bone and may be influenced by osteophytes and calcifications.

Some studies have explored using CT HU values to measure BMD, offering the advantage of separately assessing cortical and cancellous bone without interference. However, the lumbar vertebrae are different from the limb bones. Since the lumbar vertebrae are required to perform multidimensional activities, the load in the lumbar vertebrae is complex and the bone density is not uniformly distributed throughout the vertebrae. The uneven distribution of BMD in lumbar vertebrae is often overlooked in clinical practice. Therefore, the application and measurement of HU values need to be improved. In this study, the vertebral bodies were divided into anterior, middle and posterior parts to reveal the BMD characteristics of lumbar vertebrae.

### Characteristics of the distribution of HU values in the lumbar vertebrae

In this study, we grouped the patients by age and found that the HU values of the cancellous bones of the lumbar vertebrae gradually decreased with age. This decrease in bone density begins in adults around age 30 and peaks around age 50. This phenomenon is consistent with the current study showing that bone loss occurs in early adulthood after peak bone mass (PBM) is achieved at the age of 20–30 years, with peak bone loss occurring during the perimenopausal period (13, 14).

In all age groups, the HU values of the L1 and L5 vertebrae were found to be higher than those of the L2, L3, and L4 vertebrae. While this difference did not reach statistical significance, it was consistently observed across all age groups. This phenomenon may be attributed to the physiologic curvature of the lumbar spine. The Delpech-Wolff law states that bone formation is affected by mechanical stimuli. The distribution of pressure and tension shapes the microstructure of the bone and facilitates bone formation, thereby increasing its bone density, and vice versa. Due to the presence of physiologic lordosis in the lumbar spine, the pressure at L2, L3, and L4 is more skewed towards the posterior column of the vertebrae, with less stress on the anterior part of the vertebrae. Thus, the measurable HU values in L2, 3, and 4 are on the low side.

This study revealed the distribution of BMD in the lumbar spine by analyzing the HU values in anterior, middle, and posterior parts of the vertebral body. We found that BMD is not evenly distributed within the vertebrae in the lumbar spine. Specifically, HU values were found to be significantly higher in the posterior part of the L1, L2, and L3 vertebral bodies compared to the anterior part across all age groups from 20 to 79 years. However, in L4 and L5, significant differences in HU values between the anterior and posterior parts were only observed after the age of 60, with the posterior part having higher HU values.​Before 40 years of age, the HU values in the anterior and posterior parts of L4 were similar, and the HU values in the anterior part of L5 were higher than the posterior part. This may be related to the range of motion of the lumbar spine. L4-S1 has greater flexion-extension mobility than L1-L3. In young people, the anterior parts of the L4 and L5 vertebrae may be more stimulated by stress due to life and work factors, and bone formation is more active. As age increases, people over 60 years of age have less activity than younger people, and the anterior parts of L4 and L5 are not sufficiently stimulated and bone formation is attenuated.

In this study, an interesting phenomenon was found that the HU values of L1, L4 and L5 in the 70–79 age group showed abnormal increases compared with those in the 60–69 age group. The same phenomenon was also found in Wanghui’s study on the HU value of cervical vertebrae (15). The HU value of cervical vertebrae over 70 years old was higher than that of those between 60 and 70 years old. The cause of this phenomenon is not clear. Wanghui et al. suggest that the decline in HU value may reach a critical value with age. In this study, the HU values of the lumbar vertebrae were measured at the anterior, middle, and posterior parts. The results showed that the HU values of the posterior part of L1, L4 and L5 vertebral body in Group 6 were higher than those in Group 5, while the HU values of the anterior part were lower than those in Group 5. We propose that bone loss reaches a relatively stable state with aging, whereas bone remodeling is a lifelong process (16). Bone formation remained active in areas with greater stress, while bone loss continued to occur in less stressed areas, even in older people.

### Significance of lumbar HU values

#### Osteoporotic vertebral compression fractures

The occurrence of osteoporotic vertebral compression fractures (OVCFs) is prevalent among the elderly population and can significantly impact their quality of life. These fractures are primarily attributed to a decline in BMD. OVCFs often occur at the thoracolumbar junction due to the concentration of stress at this site. In this study, by comparing the CT HU values of the anterior and posterior parts of the lumbar vertebral body, it was found that the BMD of the posterior part of the L1 and L2 was significantly higher than that of the anterior part, and this pattern was consistent across all age groups. As a result, the posterior part of the vertebral body has a stronger compression resistance than the anterior part. This explains why OVCFs are more likely to occur in the anterior and middle parts of the vertebral body. OVCFs have been found to be correlated with the CT HU value of vertebrae (9), and the severity of vertebral compression is significantly correlated with the HU value (17). We believe that the uneven distribution of bone mineral density in vertebrae should be taken into account on the basis of previous studies. The HU values of the anterior and middle part of the vertebral body may be more valuable for the prediction and evaluation of the OVCFs, and the critical values still need to be further explored.

#### Cage position and cage migration

Lumbar interbody fusion (LIF) is a widely used surgery for the treatment of degenerative lumbar diseases. Major complications of LIF include cage migration, such as cage subsidence and cage retropulsion (18). The risk factors of cage subsidence after LIF included high BMI, low BMD and so on. At present, it has been confirmed that the CT HU values of vertebrae are closely related to cage subsidence (19). The HU value of the vertebrae has been utilized for the prediction of cage subsidence following lumbar surgery. In the group experiencing cage subsidence, the average HU value of the L1–4 vertebrae is approximately 116 HU (20). However, thus far, the uneven distribution of BMD in the lumbar vertebrae has not been taken into consideration by any scholars. According to current research, positioning the cage in the anterior region of the intervertebral disc spaces offers significant advantages, facilitating lumbar lordosis restoration and preventing cage retropulsion (21, 22). However, this study found that the posterior part of the vertebral body has higher BMD and stronger compression resistance than the anterior part. The mean CT HU value of the posterior part of the vertebral body in individuals aged over 70 years was observed to be more than 30 HU higher compared to that of the anterior part. We believe that the local HU value is instructive for the cage position. For special cases, such as very low BMD in the anterior part of the vertebral body, cage should be avoided in the anterior part of the disc space.

#### Pedicle screw loosening

Pedicle screw fixation is the current standard technique for the treatment of various degenerative spinal diseases and can achieve sufficient stability after the removal of most of the spinal structure. However, pedicle screw loosening is one of the major complications causing pain and decreased quality of life after spinal surgery (23). While the utilization of bone cement for reinforcing pedicle screws or expandable screws can prevent screw loosening, these measures also increase the risk of fatal cement embolization and complicate revision surgery (24). Therefore, to avoid these complications, these precautions should only be taken in patients with a very high likelihood of pedicle screw loosening (11). Average HU values of the lumbar spine have been used to predict screw loosening, and previous studies have found that HU values have better predictive value than DXA (25, 26). Jan Bredow et al. proposed that lumbar average HU values below 120HU could be a risk factor for pedicle screw loosening (27). However, no studies have taken into account the uneven distribution of vertebral BMD. Biomechanical studies have demonstrated that bones located in the pedicle region and in the posterior part of the vertebrae play a more significant role in enhancing the stability of the pedicle screw, particularly in the presence of low BMD (28). Our study suggests that older adults have a higher BMD at the posterior part of the vertebral body, which may provide greater control force for the pedicle screw. Therefore, for the surgeon, the CT HU value of the implantation area of the pedicle screw may have a higher value than the CT HU value of the entire vertebra for predicting the stability of the pedicle screw and for making surgical plans.

### Limitations

There are some limitations to the current study. The load distribution on the spine is largely determined by its curvature on the sagittal plane (29). Gustaw Wojcik et al. proposed that the lumbar lordosis Angle causes changes in pressure and shear forces, which can lead to changes in BMD (30). In this study, the included data came from patients attending our hospital and the lumbar lordosis (LL) values may be affected by factors such as pain. Therefore, the correlation between LL and lumbar BMD distribution was not included in this study. The effect of lumbar curvature on the distribution of BMD in the lumbar spine needs to be investigated further in the future. In addition, the correlation between HU values in different parts of the vertebrae and DXA results still needs to be further explored in future studies. Finally, the data we collected were from patients with degenerative lumbar disease, and it remains to investigate whether the same trend exists in the normal population.

## Data Availability

The raw data supporting the conclusions of this article will be made available by the authors, without undue reservation.

## References

[1] Kanis JA Melton LJ 3rd Christiansen C Johnston CC Khaltaev N. The diagnosis of osteoporosis. J Bone Miner Res. (1994) 9:1137–41. doi: 10.1002/jbmr.5650090802 7976495

[2] Schreiber JJ Anderson PA Rosas HG Buchholz AL Au AG. Hounsfield units for assessing bone mineral density and strength: a tool for osteoporosis management. J Bone Joint Surg Am. (2011) 93:1057–63. doi: 10.2106/JBJS.J.00160 21655899

[3] Lee S Chung CK Oh SH Park SB. Correlation between bone mineral density measured by dual-energy X-ray absorptiometry and hounsfield units measured by diagnostic CT in lumbar spine. J Korean Neurosurg Soc. (2013) 54:384–9. doi: 10.3340/jkns.2013.54.5.384 PMC3873350 24379944

[4] Silva IM Freitas DQ Ambrosano GM Bóscolo FN Almeida SM. Bone density: comparative evaluation of Hounsfield units in multislice and cone-beam computed tomography. Braz Oral Res. (2012) 26:550–6. doi: 10.1590/s1806-83242012000600011 23184166

[5] Mi J Li K Zhao X Zhao CQ Li H Zhao J. Vertebral body compressive strength evaluated by dual-energy X-ray absorptiometry and hounsfield units in vitro. J Clin Densitom. (2018) 21:148–53. doi: 10.1016/j.jocd.2016.08.011 27623115

[6] Zou D Jiang S Zhou S Sun Z Zhong W Du G. Prevalence of osteoporosis in patients undergoing lumbar fusion for lumbar degenerative diseases: A combination of DXA and hounsfield units. Spine (Phila Pa 1976). (2020) 45:E406–10. doi: 10.1097/BRS.0000000000003284 31725127

[7] Choi MK Kim SM Lim JK. Diagnostic efficacy of Hounsfield units in spine CT for the assessment of real bone mineral density of degenerative spine: correlation study between T-scores determined by DEXA scan and Hounsfield units from CT. Acta Neurochir (Wien). (2016) 158:1421–7. doi: 10.1007/s00701-016-2821-5 27177734

[8] Zou D Li W Deng C Du G Xu N. The use of CT Hounsfield unit values to identify the undiagnosed spinal osteoporosis in patients with lumbar degenerative diseases. Eur Spine J. (2019) 28:1758–66. doi: 10.1007/s00586-018-5776-9 30306332

[9] Zou D Ye K Tian Y Li W Zhou F Zhang Z. Characteristics of vertebral CT Hounsfield units in elderly patients with acute vertebral fragility fractures. Eur Spine J. (2020) 29:1092–7. doi: 10.1007/s00586-020-06363-1 32157387

[10] Pisano AJ Fredericks DR Steelman T Riccio C Helgeson MD Wagner SC. Lumbar disc height and vertebral Hounsfield units: association with interbody cage subsidence. Neurosurg Focus. (2020) 49:E9. doi: 10.3171/2020.4.FOCUS20286 32738808

[11] Li W Zhu H Hua Z Miao D Wang F Tong T. Vertebral bone quality score as a predictor of pedicle screw loosening following surgery for degenerative lumbar disease. Spine (Phila Pa 1976). (2023) 48:1635–41. doi: 10.1097/BRS.0000000000004577 PMC10624406 36728017

[12] Muraki S Yamamoto S Ishibashi H Horiuchi T Hosoi T Orimo H. Impact of degenerative spinal diseases on bone mineral density of the lumbar spine in elderly women. Osteoporos Int. (2004) 15:724–8. doi: 10.1007/s00198-004-1600-y 14997287

[13] Rozenberg S Bruyère O Bergmann P Cavalier E Gielen E Goemaere S. How to manage osteoporosis before the age of 50. Maturitas. (2020) 138:14–25. doi: 10.1016/j.maturitas.2020.05.004 32631584

[14] Khosla S. Pathogenesis of age-related bone loss in humans. J Gerontol A Biol Sci Med Sci. (2013) 68:1226–35. doi: 10.1093/gerona/gls163 PMC3826857 22923429

[15] Liang X Liu Q Xu J Ding W Wang H. Hounsfield unit for assessing bone mineral density distribution within cervical vertebrae and its correlation with the intervertebral disc degeneration. Front Endocrinol (Lausanne). (2022) 13:920167. doi: 10.3389/fendo.2022.920167 35872993 PMC9304988

[16] Kenkre JS Bassett J. The bone remodelling cycle. Ann Clin Biochem. (2018) 55:308–27. doi: 10.1177/0004563218759371 29368538

[17] Li C Lai XM Liu N Lin Y Hu W. Correlation analysis of the vertebral compression degree and CT HU value in elderly patients with osteoporotic thoracolumbar fractures. J Orthop Surg Res. (2023) 18:457. doi: 10.1186/s13018-023-03941-z 37365576 PMC10294538

[18] Chen L Yang H Tang T. Cage migration in spondylolisthesis treated with posterior lumbar interbody fusion using BAK cages. Spine. (2005) 30:2171–5. doi: 10.1097/01.brs.0000180402.50500.5b 16205342

[19] Mi J Li K Zhao X Zhao CQ Li H Zhao J. Vertebral body hounsfield units are associated with cage subsidence after transforaminal lumbar interbody fusion with unilateral pedicle screw fixation. Clin Spine Surg. (2017) 30:E1130–6. doi: 10.1097/BSD.0000000000000490 27906743

[20] Xie F Yang Z Tu Z Huang P Wang Z Luo Z. The value of Hounsfield units in predicting cage subsidence after transforaminal lumbar interbody fusion. BMC Musculoskelet Disord. (2022) 23:882. doi: 10.1186/s12891-022-05836-2 36138360 PMC9502605

[21] Landham PR Don AS Robertson PA. Do position and size matter? An analysis of cage and placement variables for optimum lordosis in PLIF reconstruction. Eur Spine J. (2017) 26:2843–50. doi: 10.1007/s00586-017-5170-z 28620787

[22] Hu YH Niu CC Hsieh MK Tsai TT Chen WJ Lai PL. Cage positioning as a risk factor for posterior cage migration following transforaminal lumbar interbody fusion - an analysis of 953 cases. BMC Musculoskelet Disord. (2019) 20:260. doi: 10.1186/s12891-019-2630-0 31142310 PMC6542074

[23] Banno T Hasegawa T Yamato Y Yoshida G Arima H Oe S. The incidence of iliac screw-related complications after long fusion surgery in patients with adult spinal deformity. Spine (Phila Pa 1976). (2022) 47:539–47. doi: 10.1097/BRS.0000000000004276 34798648

[24] Mo GY Zhou TP Guo HZ Li YX Tang YC Guo DQ. Long-term efficacy and safety of bone cement-augmented pedicle screw fixation for stage III Kümmell disease. Sci Rep. (2021) 11:13647. doi: 10.1038/s41598-021-93013-1 34211025 PMC8249396

[25] Kim KH Kim TH Kim SW Kim JH Lee HS Chang IB. Significance of measuring lumbar spine 3-dimensional computed tomography hounsfield units to predict screw loosening. World Neurosurg. (2022) 165:e555–62. doi: 10.1016/j.wneu.2022.06.104 35772704

[26] Zou D Sun Z Zhou S Zhong W Li W. Hounsfield units value is a better predictor of pedicle screw loosening than the T-score of DXA in patients with lumbar degenerative diseases. Eur Spine J. (2020) 29:1105–11. doi: 10.1007/s00586-020-06386-8 32211997

[27] Bredow J Boese CK Werner CM Siewe J Löhrer L Zarghooni K. Predictive validity of preoperative CT scans and the risk of pedicle screw loosening in spinal surgery. Arch Orthop Trauma Surg. (2016) 136:1063–7. doi: 10.1007/s00402-016-2487-8 27312862

[28] Cornaz F Farshad M Widmer J. Location of pedicle screw hold in relation to bone quality and loads. Front Bioeng Biotechnol. (2022) 10:953119. doi: 10.3389/fbioe.2022.953119 36118575 PMC9478651

[29] Yilgor C Sogunmez N Boissiere L Yavuz Y Obeid I Kleinstück F. Global alignment and proportion (GAP) score: development and validation of a new method of analyzing spinopelvic alignment to predict mechanical complications after adult spinal deformity surgery. J Bone Joint Surg Am. (2017) 99:1661–72. doi: 10.2106/JBJS.16.01594 28976431

[30] Wojcik G Rutkowska E Mysula I Szepeluk A. Lumbar lordosis angle value analysis and bone tissue density in the ls section in women after 50 years old. Wiad Lek. (2020) 73:708–14. doi: 10.36740/wlek202004116 32731702

