# Corrigendum: Hounsfield unit for assessing bone mineral density distribution within lumbar vertebrae and its clinical values

**DOI:** 10.3389/fendo.2025.1568596

**Published:** 2025-02-14

**Authors:** Jiabao Chen, Yanhong Li, Han Zheng, Haotian Li, Haidong Wang, Lei Ma

**Affiliations:** ^1^ Department of Spinal Surgery, The Third Hospital of Hebei Medical University, Shijiazhuang, China; ^2^ Department of Internal Medical, Hebei Medical University, Shijiazhuang, China

**Keywords:** Hounsfield unit, bone mineral density distribution, lumbar vertebrae, osteoporosis, osteoporotic vertebral compression fractures, lumbar surgery

In the published article, there was an error in the **Funding** statement. The project number that funded this research failed to be mentioned in the article. The correct **Funding** statement appears below.

“The author(s) declare financial support was received for the research, authorship, and/or publication of this article. This study was supported by Hebei Natural Science Foundation (H2020206379) and Hebei Province medical technology tracking project (GZ2024051).”

The authors apologize for this error and state that this does not change the scientific conclusions of the article in any way. The original article has been updated.

